# Desidustat: a novel PHD inhibitor for the treatment of CKD-induced anemia

**DOI:** 10.3389/fneph.2024.1459425

**Published:** 2024-10-22

**Authors:** Amit Joharapurkar, Vrajesh Pandya, Harilal Patel, Mukul Jain, Ranjit Desai

**Affiliations:** Zydus Research Centre, Zydus Lifesciences Limited, Ahmedabad, Gujarat, India

**Keywords:** prolyl hydroxylase inhibitor, HIF, chronic kidney disease-induced anemia, hepcidin, hemoglobin

## Abstract

Desidustat is a small molecule inhibitor of hypoxia-inducible factor-prolyl hydroxylase (HIF-PH) discovered and developed by Zydus Lifesciences for the treatment of anemia associated with chronic kidney disease (CKD). This review summarizes the preclinical and clinical profile of desidustat which led to its approval and clinical use in India.

## 1 Introduction

### 1.1 Anemia of chronic kidney disease

Anemia (defined as hemoglobin [Hb]<11 g/dL in women and<12 g/dL in men) has a prevalence of <10% in stages I & II, about 20–40% in stage III, and 50–60% in stage IV of CKD. This prevalence even exceeds 70% in stage V due to the decline in estimated glomerular filtration rate (eGFR) (1). Global prevalence of CKD was estimated to be 11% to 14% in the first two decades of twenty-first century (2). Hemoglobin (Hb) and red blood cells (RBC) deficiency observed in CKD is caused due to a combination of factors like deficiency in iron utilization, relative erythropoietin (EPO) deficiency, and resistance to the actions of erythropoietin (3). Systemic inflammation, uremia, and vitamin deficiency also contribute to the progression of CKD-induced anemia. The severity of anemia has a direct correlation with progression of chronic kidney disease (decrease in eGFR) and morbidity due to cardiorenal diseases (4). Anemia is also observed in diabetic patients, independent of eGFR and albuminuria (3).

### 1.2 Regulation of RBC synthesis by erythropoietin

EPO is synthesized and secreted by the fibroblast-like interstitial peritubular cells of the kidneys, and the perisinusoidal cells of the liver may also secrete EPO in a minor fashion (5). In bone marrow, the hematopoietic stem cells (HSCs) differentiate into colony forming units-erythroid (CFU-Es) and then to erythroblasts, which form reticulocytes by enucleation. This process requires stimulation by EPO. Erythrocytes or RBC are formed from reticulocytes within one or two days, which circulate in the blood for 120 days, following which, they are killed by phagocytosis in the spleen and liver (6). EPO acts on specific EPO receptors (EPORs) which signal through Janus tyrosine kinase-2 (JAK2). JAK2 stimulates signal transduction and activator of transcription-5 (STAT-5), RAS−RAF−MAP kinase, and phosphoinositide-3 kinase/AKT kinase (protein kinase B). On the other hand, erythroid progenitors undergo apoptosis by the cluster of differentiation 95 (CD95), a membrane protein of the tumor necrosis factor (TNF) receptor family, which triggers apoptosis after binding to the CD95 ligand, which is produced by mature erythroblasts. Thus, a negative feedback cycle controls the production of mature erythrocytes in blood (7).

### 1.3 Role of iron and hepcidin in RBC synthesis

Apart from erythropoietin, which differentiates the erythrocyte precursors, iron is the next important contributor in efficient synthesis of hemoglobin and RBC synthesis by reticulocyte maturation is iron.

Iron from diet is absorbed in the duodenum and proximal jejunum, when it is in the ferrous (Fe2+) state or bound by a protein such as heme. Duodenal cytochrome B (Dcytb), on the brush border of the enterocytes converts the insoluble ferric (Fe3+) to absorbable ferrous (Fe2+) ions and divalent metal cation transporter 1 (DMT1) transports ferrous (Fe2+) iron in to the cell. The cellular iron is transported in the circulation by the transmembrane efflux protein ferroportin. Ferrous iron (Fe2+) is oxidized (by ceruloplasmin and hephaestin) into ferric iron (Fe3+) for binding to transferrin, which serves as the iron-carrier protein in plasma. Transferrin chelates iron to make it soluble, prevents the formation of reactive oxygen species, and facilitates its transport into cells.

Iron metabolism is monitored by the hepatic hormone hepcidin, which exclusively regulates the transporter ferroportin. Inflammatory states increase cytokine (e.g., IL-6) release, which stimulates hepcidin expression in the liver. Hepcidin causes decreased iron absorption through ferroportin degradation and decreases the release of iron from macrophages (8). In addition to hepcidin, levels of DMT1 and Dcytb are upregulated in the hypoxic environment of the intestinal mucosa by hypoxia-inducible factor-2 (HIF-2α) (9). High levels of iron, inflammatory cytokines, and oxygen lead to increased levels of hepcidin. Hepcidin binds to ferroportin, resulting in its internalization and degradation and effectively shunting cellular iron into ferritin stores and preventing its absorption into the blood. Thus, increased hepcidin traps body iron in macrophages, hepatocytes, and enterocytes. Thereby, hepcidin potentiates the excretion of iron through the sloughing of enterocytes (and their ferritin stores) into the feces and out of the body.

### 1.4 Limitations of the use of erythropoietin and iron in treatment of CKD-induced CKD

The treatment of CKD-induced anemia is done primarily using recombinant EPO (10). However, in end-stage renal disease and in nondialysis chronic kidney disease patients, treatment with EPO or ESA (erythropoiesis stimulating agents) showed inadequate efficacy and cardiovascular toxicity in terms of risk of cardiovascular hospitalization or stroke (11). The risks due to EPO treatment can be correlated with inflammatory markers and urinary protein/creatinine ratio (12). Earlier, the increase in Hb and blood volume or viscosity thereby was considered the reason for cardiovascular disease. However, it is now evident that elevation of Hb into the normal range in a spontaneous way is not detrimental, but rather beneficial for cardiovascular health (13). ESA treatment, either supraphysiological or after prolonged exposure, also results into ESA resistance which is associated with the disease progression, infection and inflammation, increased cytokine signaling, and defective iron metabolism (14). On the other hand, it has been demonstrated that improving ESA sensitivity reduces the cardiovascular toxicity of the erythropoietin preparations (15), and higher doses of EPO can induce morbidity and mortality (16). A sizable population of CKD patients require higher doses of EPO, which may culminate into EPO resistance and cardiovascular morbidities (17). Anti-EPO antibodies are generated in some CKD patients after treatment with ESA and it is related with EPO resistance, resulting in worsening of the anemia (18). EPO antibodies mediate pure red cell aplasia after treatment with recombinant EPO products.

In CKD patients, body iron regulation is disturbed, with increased iron loss, decreased iron absorption, and limited iron availability leading to iron deficiency. Intravenous (IV) iron therapy is frequently prescribed to replace lost iron, which eventually causes rise in hepcidin levels and traps iron in the tissue stores. Also, the increased iron in circulation imposes toxicity without effective erythropoiesis (19).

### 1.5 HIF system: an endogenous regulator of efficient erythropoiesis

High altitude or hypoxia enhances RBC synthesis by stimulating EPO production by induction of hypoxia-inducible factor (HIF) (20). There are three isoforms of HIF (HIF-1, -2, and -3). HIF-1 mainly regulates the metabolic response, while HIF-2 stimulates erythropoiesis in response to hypoxia. HIF-3 is less closely related to HIF-1 and HIF-2, and its role is not yet fully understood. HIF consists of two subunits, namely α and β. HIF-α is hydroxylated at two proline residues (Pro402 and Pro564 for HIF-1α, and Pro405 and Pro531 for HIF-2α) by prolyl hydroxylase domain enzyme (PHD). Once hydroxylated, HIF-α is recognized by Hippel−Lindau tumor suppressor protein (pVHL), which causes its proteasomal degradation by E3 ubiquitin ligase. PHD enzymes require oxygen, iron, ascorbate, and the substrate 2-oxoglutarate (2- OG) for their activity. Oxygen deficiency (hypoxia) causes PHD inhibition, which activates HIF-α, while HIF-β is constitutively expressed.

There are three isoforms of PHD, namely, PHD1, PHD2, and PHD3 (21). The active sites of all isoforms of PHDs share a high-sequence homology. PHD1 shows more affinity toward HIF-2α than HIF-1α and hydroxylates it under normoxia condition. Deletion of the PHD1 gene induces hypoxia tolerance by shifting the tricarboxylic acid cycle to the glycolytic pathway (22). Tolerance to hypoxia is due to reduced oxidative stress and may not be due to angiogenesis, erythropoiesis, or vasodilation. PHD2 gene deletion accelerated RBC synthesis without a significant increase in EPO levels (23). Inactivation of PHD1 and PHD3 leads to erythrocytosis by activating the hepatic HIF-2α, whereas only PHD2 deficiency leads to erythrocytosis by activating the renal EPO pathway (21).

### 1.6 Involvement of HIF in fibrosis and iron metabolism

Interstitial fibroblasts of kidneys generate EPO (24). Fibrosis in the renal tissue causes EPO deficiency and anemia. HIF-2 stimulates EPO gene transcription by binding to hypoxia-responsive regulatory elements. HIF-2 induces efficient absorption as well as utilization of iron by stimulation of genes required for iron transport and metabolism like DMT1 and Dcytb. HIF indirectly stimulates efficient iron utilization through regulation of hepcidin (25). Inflammation upregulates hepcidin through the STAT-3 pathway or the BMP/SMAD1/5/8 pathway (19, 25). As already discussed, increased hepcidin causes degradation of ferroportin and causes entrapment of iron in tissue stores, resulting in a functional iron deficiency, which cannot be corrected using oral iron supplementation. As the iron deficiency worsens, iron regulatory proteins bind to HIF- 2α and further decrease erythropoiesis (26). This feedback mechanism makes the anemia in CKD patients difficult to treat using erythropoietin analogues or iron supplementation.

### 1.7 HIF-PHD inhibitors: a novel way to correct CKD-induced anemia

**Figure 1:**
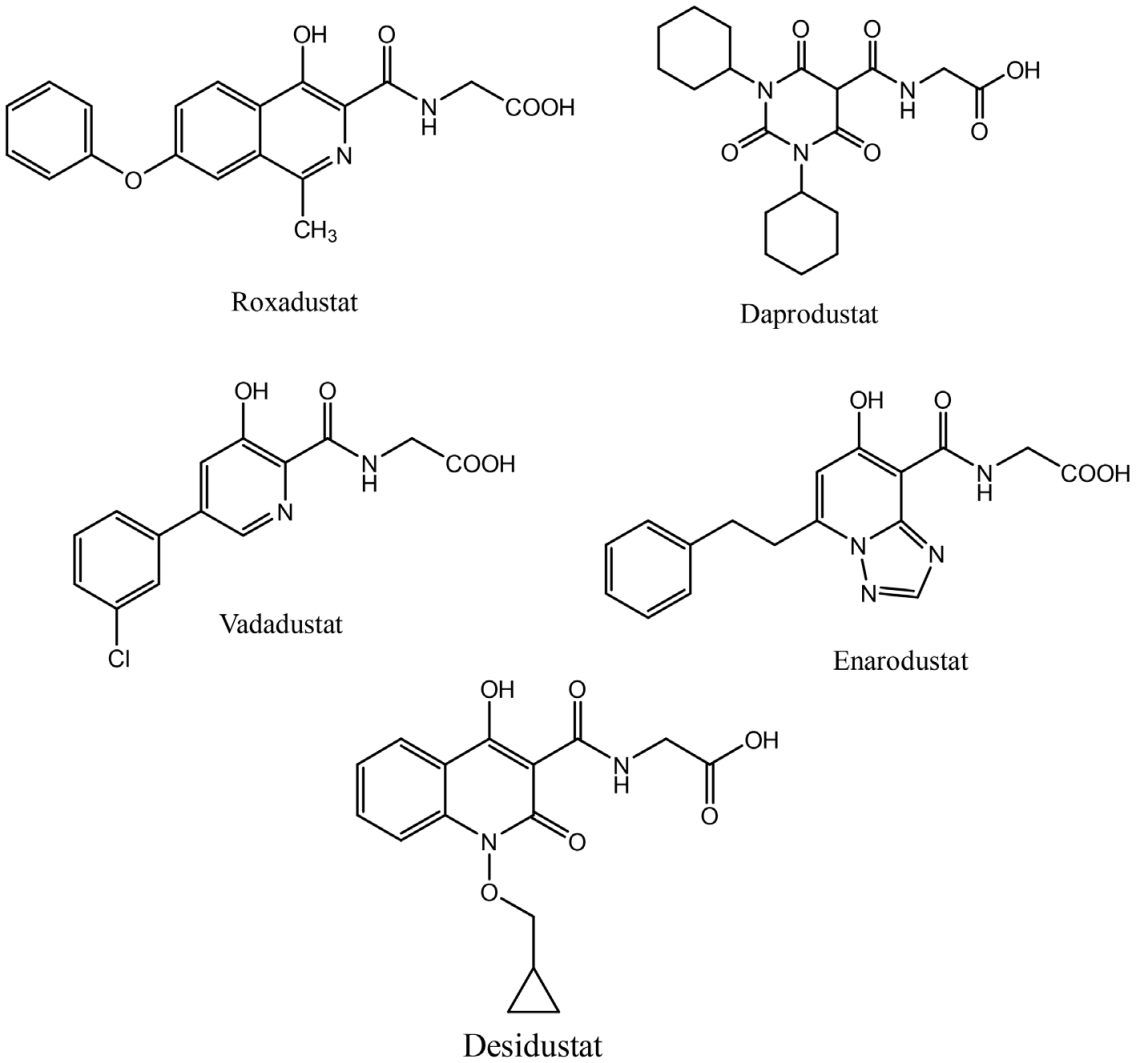
Selected PHD inhibitors.

Activation of HIF signaling using PHD inhibition is a unique and rational approach to correct anemia associated with chronic diseases. Desidustat is a novel HIF-PHD inhibitor discovered and developed by Zydus Lifesciences for the treatment of anemia in patients with CKD patients on dialysis and also those who are not on dialysis. Desidustat got its first approval in India in 2022 (27). Currently, PHD inhibitors are approved in different countries. Roxadustat, the first clinically used PHD inhibitor, is approved in a number of countries, including China, Japan, Chile, South Korea, and Europe for the treatment of anemia in CKD in NDD and DD adult patients. However, it was not approved in USA for treatment of anemia of CKD (both NDD and DD patients), because a higher portion of NDD-CKD patients and DD-CKD patients experienced vascular access thrombosis compared with controls (28). Recently, vadadustat and daprodustat have been approved for clinical use in USA in addition to their previous approvals in Japan and China. In US, vadadustat and daprodustat are approved by FDA only for dialysis patients who have been receiving dialysis at least for four months. Few other HIF-PHD inhibitors, molidustat (Japan and China) and enarodustat (Japan) have also found their way to the market. These HIF-PHD inhibitors (**Figure 1**) have met their primary goal of correcting anemia in CKD patients, however, these molecules demonstrated differential profile in terms of efficacy and toxicity.

### 1.8 Pleiotropic effects of HIF stabilization

Clinically approved HIF-PHD inhibitors are the functional inhibitors of 2-oxoglutarate, which is a substrate of PHD (29). Since the pathways controlled by 2-oxoglutarate cover a diverse range of physiological processes, inhibition of PHD may affect many genes that are unrelated to anemia, whose protein products exert important functions. The genes that are modulated by HIF but may be unrelated to erythropoiesis (or anemia) include those related to glucose metabolism, energy homeostasis, angiogenesis, migration and interaction of cells and many other metabolic pathways. The modulation of such genes by HIF-PHD inhibitors may cause the complications like cancer progression, retinal diseases, pulmonary hypertension, and cyst formation in polycystic kidney diseases in adults (30), due to the downstream pathways like induction of vascular endothelial growth factor, proliferation of endothelial colony-forming cells, or chloride-dependent fluid secretion in case of cysts (30, 31). However, these safety concerns were not found to be significant in the clinical trials of the HIF-PHD inhibitors so far.

The transient and frequent PHD inhibition caused by the HIF-PHD inhibitors may also enhance the production of reactive oxygen species since the switch of metabolism from aerobic to anaerobic glycolysis (32). HIF stabilization modulate osteogenic differentiation through the expression of RUNX2 gene, and HIF-PHD inhibitors may cause vascular calcification (30). Daprodustat, a HIF-PHD inhibitor caused vascular calcification in a mice model of CKD and hyperphosphatemia, which was due to stabilization of HIF (33). Clinical studies with a long follow-up period are needed to evaluate the possible risk of clinically relevant calcification in CKD patients with hyperphosphatemia.

### 1.9 Thrombotic and cardiac effects of HIF-PHD inhibition

HIF-PHD inhibitor treatment may also cause thromboembolism, roxadustat caused more thrombosis than ESAs and vadadustat, according to an analysis, and the lowest rates of thrombosis were seen with molidustat and ESAs, with the US FDA raising concern for thrombotic risk of certain HIF-PHD inhibitors (34). Thrombosis risk is increased by HIF-PHD inhibitor treatment because they can modulate the tissue factors involved in thrombosis or protein kinase B that contributes to platelet and monocyte activation (30). HIF is also partially responsible for the remodeling of heart leading to worsening of heart failure (35). The use of HIF-PHD inhibitor daprodustat was associated with an increase in the estimated hazard ratio (HR) for hospitalization for heart failure in patients with a history of heart failure in patients not undergoing dialysis (36). It is possible that administration of HIF-PHD inhibitors to patients with heart failure causes an imbalance between the energy demand and supply in heart, leading to cardiac hypertrophy.

Most of the phase 3 studies with HIF-PHD inhibitors were done with strict exclusion criteria for patients with comorbidities of cardiac metabolism. Hence, post-marketing trials would be needed to ensure the safety of HIF-PHD in clinical use.

## 2 Desidustat, a novel PHD inhibitor

### 2.1 Mechanism of action of desidustat

The *in vitro* activity of desidustat was assessed in two HIF heterodimerization bioassays which measured stabilization of HIF in live cells and in real time (37). EC_50_ and E_max_ values were determined as a measure of relative potency and efficacy, respectively. Desidustat achieved maximum stabilization of both, HIF1α and HIF2α. In HIF1 and 2 bioassays, desidustat was found to be a HIF stabilizer (EC_50_ = 32.6 μM for HIF1α and 22.1 μM for HIF2α), while it showed the optimum efficacy (E_max_ = 119% in both HIF1α and HIF2α assays). In this assay, roxadustat showed similar activity as that of desidustat with 105% and 98.9% Emax at micromolar concentrations. However, daprodustat was comparatively weaker, since it could show only 37.9% and 51.8% Emax. Desidustat stabilizes HIF in liver as well as kidneys of rats, after single dose administration (37).

### 2.2 Desidustat increases serum erythropoietin, reticulocyte rise, and also improves erythropoiesis in normal rats

Desidustat caused a dose-related increase in serum erythropoietin when dosed from 5 mg/kg to 60 mg/kg oral dose, and also caused a dose-related increase in reticulocyte count. In these rats, the HIF stabilization caused by desidustat was also associated with transient increase in serum iron which was normalized within 48 days of dosing. After repeated dosing (alternate day for a week), the lowest dose of 5 mg/kg caused an increase of 0.9 g/dl in hemoglobin in these rats, which increased in a dose-related way up to the 60 mg/kg doses tested. These doses caused a dose-related increase in erythropoietin, but not in serum Vascular Endothelial Growth Factor (VEGF) (38).

### 2.3 Desidustat is effective in treating anemia in rats after five-sixth nephrectomy

The erythropoietic activity of roxadustat was determined in a rat model of anemia of CKD induced by five-sixth nephrectomy. The nephrectomized rats showed a significant loss in hemoglobin, RBC count and hematocrit, compared to the sham-treated animals. These nephrectomized animals were treated with desidustat on alternate day with the dose ranging from 05 mg/kg to 60 mg/kg by oral route, with darbepoietin (16 µg/kg, subcutaneous, once a week) as the comparator for 28 days. Starting from dose of 15 mg/kg, desidustat showed a significant and dose-related improvement in hemoglobin, RBC count and hematocrit, the serum iron was also increased but it normalized with 24 hours of last dose. The increases in the hemoglobin and hematocrit showed by desidustat at 15 mg/kg dose were comparable to those shown by darbepoietin. In addition, desidustat treatment caused a meaningful reduction in liver hepcidin after twenty-eight days of the treatment (38).

### 2.4 Desidustat corrects anemia of inflammation in mice

Chronic inflammatory diseases are often associated with anemia, and treatment with erythropoiesis stimulating agents (ESAs) are associated with potentially hazardous side effects and poor outcomes. In rodent models of inflammation, desidustat was assessed as an effective treatment of anemia of inflammation. In BALB/c mice, a single dose treatment of desidustat (15mg/kg, orally) attenuated the effect of lipopolysaccharide (LPS) - or turpentine oil-induced inflammation and increased serum erythropoietin (EPO), iron, and reticulocyte count, and decreased serum hepcidin levels, in a dose related manner (39).

### 2.5 Desidustat improves the anemia of chronic disease in rats

The model of anemia of chronic disease (ACD) was established by administration of proteoglycan-polysaccharide (PG-PS). Desidustat can reverse the anemia caused by the PG-PS stimulus, which induces inflammation and functional iron deficiency. In female Lewis rats, treatment with desidustat (15 and 30 mg/kg, orally) markedly reduced PGPS-induced anemia and increased hemoglobin, red blood cell (RBC) and white blood cell (WBC) count, hematocrit, serum iron and spleen iron. These effects of desidustat were associated with reduction in hepcidin gene (HAMP) expression as well as reduction in serum hepcidin, and increased EPO expression in liver and kidneys. Desidustat increased the expression of duodenal cytochrome B (DcytB), ferroportin (FPN1) and divalent metal transporter 1 (DMT1) in duodenum, and FPN1 and monocyte chemoattractant protein-1 (MCP-1) in liver suggesting an overall influence on iron metabolism (39).

### 2.6 Desidustat is useful in treating chemotherapy-induced anemia

Cisplatin administration in mice causes the decrease in hemoglobin, hematocrit, and RBC count. Oral treatment with desidustat (15 mg/kg and 30 mg/kg, alternate day) for twenty-eight days caused a dose-related increase in all these three parameters (38).

### 2.7 Desidustat reduces acute and chronic kidney injury by reducing fibrosis and inflammation in rats

Studies in SD rats and C57 mice (38–40) studies indicate that 15 mg/kg on alternate day dose is the efficacious dose for improvement in hemoglobin, in anemia induced by nephrectomy or inflammation. Desidustat reduces alternate compliment pathways through partial inhibition of factor B, which is useful in treatment of immune-mediated renal and hematological disorders (41). Desidustat prevents acute kidney injury in rats induced by ischemia (42). Treatment with desidustat reversed the decrease in serum erythropoietin caused by the ischemia injury and prevented the increase in serum creatinine, urea, and KIM-1-the known markers of kidney injury. Desidustat treatment caused a significant increase in serum erythropoietin levels, which was accompanied by reduction in serum IL-6 and IL-1β levels. Thus, desidustat treatment can improve erythropoietin by a combination of direct HIF-stabilizing effect and reduction in the proinflammatory cytokines (42). Adenine converts into 2, 8-dihydroxyadenine in the body and deposits as crystals in the proximal tubular epithelia of the kidney, which induces inflammation and subsequent tubulointerstitial fibrosis and anemia (43). Desidustat treatment effectively reduced the adenine-induced nephritis in mice as indicated by reduced creatinine and urea, and reduced the urinary albumin to creatinine ratio. This treatment caused a profound decrease in the inflammatory milieu induced by the adenine supplementation with decreased IL-1 β and IL-6 levels in both kidney and serum. Also, a significant reduction in liver hepcidin and renal myeloperoxidase indicated by HIF stabilization and reduction in oxidative stress associated fibrosis (42).

### 2.8 Desidustat improves erythropoietin resistance in anemic state in rats

Though erythropoietin therapy is the mainstay of CKD-induced anemia, a significant CKD patients are refractory to erythropoietin (EPO) effects due to inflammation, deranged iron utilization, and generation of EPO antibodies (14). Effect of desidustat on EPO-refractory renal anemia was assessed in Sprague Dawley rats that were made anemic by cisplatin (5 mg/kg, IP, single dose) and turpentine oil (5 mL/kg, SC, once a week). These rats were given recombinant human EPO (rhEPO, 1 μg/kg) and desidustat (15 or 30 mg/kg) for eight weeks. Separately, rhEPO (1–5 μg/kg) was given to anemic rats to sustain the normal hemoglobin levels and desidustat (15 mg/kg) for eight weeks. In another experiment, the anemic rats were treated rhEPO (5 μg/kg) for two weeks and then desidustat (15 mg/kg) for the next two weeks. Dosing of rhEPO was thrice a week, and for desidustat, it was on alternate days. Desidustat inhibited EPO-resistance caused by rhEPO treatment, decreased hepcidin, IL-6, IL-1β, and increased iron and liver ferroportin. Desidustat reduced EPO requirement and anti-EPO antibodies. Desidustat also maintained normal hemoglobin levels after cessation of rhEPO treatment. Thus, novel prolyl hydroxylase inhibitor desidustat can treat EPO resistance via improved iron utilization and decreased inflammation (10).

### 2.9 Preclinical pharmacokinetics of desidustat

Desidustat (15 mg/kg, oral) administration in rats is rapidly absorbed with Tmax of 1.0 h, with Cmax of 10.48µg/ml in the blood. The peak erythropoietin concentration was observed at 6 h after the dose, and the resultant increase in reticulocyte and hemoglobin-RBC indicated an alternate day dosing for an optimum efficacy (38, 40). The clearance of the compound is moderate, with t1/2 of 6.35 h. The major route of excretion was found to be the renal route, with 39% of unchanged drug excreted in the urine. However, the lack of clinically meaningful effect on systemic clearance of desidustat, even after 5/6th removal of kidneys or complete removal of the two kidneys in rats, suggest the importance of non-renal clearance mechanism on the overall disposition of desidustat (40).

## 3 Clinical studies of desidustat

### 3.1 Safety and pharmacokinetics of desidustat

In single-dose studies, desidustat was well-tolerated up to 300 mg/kg in mice, up to 800 mg/kg in rats, and up to 75 mg/kg in beagle dogs by the oral route. Based on one-month toxicity studies, 60 mg/kg in rats and 15 mg/kg in dogs was considered the maximum tolerated dose (MTD) and 5 mg/kg was considered the no observed adverse effect level (NOAEL) in the 28-day toxicity studies in dogs (44). The toxicity signs were mainly associated with accelerated pharmacology, related to the hemoglobin build-up in the blood, and it was reversible.

Phase I study was a randomized, double-blind, placebo-controlled trial, conducted in addition to a third part with open-label to evaluate the food/sex effect (44). Fifty six participants were enrolled in the first part, where a single dose of desidustat with strengths of 10, 25, 50, 100, 150, 200, and 300 mg was given to the volunteers. In the second part, every day single dose of 100, 150, 200, and 300 mg was given to thirty two participants. The gender and food effect were studied in twelve participants with 150 mg dose of desidustat.

The Cmax was 566.4, 6745, and 17858.3 ng/ml at 10 mg, 100 mg and 300 mg dose, with a dose-related pattern, and the AUC (h.ng/ml) for 24 hours was found to be 3597.2, 46,844.7, and 115,898.8, respectively for these doses. The tmax mean was found to be 1.25 to 3 h at this dose range, and the t1/2 mean range was 6.9 to 11.3 hours. Similar to the preclinical pharmacokinetic study, the excretion of drug was found in urine, with 27.5 to 41.1% drug excreted unchanged in the urine. The data indicated no accumulation of the drug at 100, 150, 200, and 300 mg doses.

Absorption of desidustat was delayed in presence of food (median tmax was 2 h as against 5 h in fed condition, with Cmax decreased by 2.39 fold in presence of food). However, the half-life of the drug was similar in fed or fasted conditions. The mean Cmax for male and female subjects were not different, though the extent of absorption in males was more than in females.

### 3.2 Desidustat showed erythropoietic response in healthy subjects

Desidustat showed a dose-related increase in mean serum EPO levels from 100 mg to 300 mg dose, with mean serum EPO Cmax values ranging from 6.6 mIU/L at 10 mg to 79.9 mIU/L at 300 mg dose level. The EPO level peak in placebo-treated subjects was 9.6 mIU/L, and in all subjects, the EPO levels returned to the baseline within 24 hours of the single dose administration. The serum EPO profile after multiple dosing followed a similar profile to single dosing, with a lag in time of approximately 6–8 h to EPO Cmax from pharmacokinetic tmax for desidustat. A higher number of subjects who received desidustat showed a hemoglobin increase of >0.5 g/dl at any timepoint compared to the placebo group. Also, the hemoglobin increase of >1.0 g/dl was seen more in subjects treated with desidustat, when compared to those that received placebo.

### 3.3 Desidustat was safe with no serious adverse events in phase 1 trials

The drug-related adverse events (AEs) were of grade 1 severity in the single dose study and they included dizziness and headache were the most common symptoms. In the multiple dose study, the treatment-related AEs were all grade 1 in severity, with the exception of dizziness in two subjects, which was classified as grade 2 in severity (44).

### 3.4 Clinical trials of desidustat

#### 3.4.1 Desidustat shows dose-related efficacy comparable to standard of care without accumulation in phase 2 trial

Phase 2 trial of desidustat was a placebo-controlled, randomized, double-blind, parallel group and multicentric study (45), Desidustat doses were 100, 150, and 200 mg, administered alternate day for 6 weeks. The mean age of the patients was 48 years, with 57% females, and 43% male patients, and their mean baseline hemoglobin was 9.44 g/dL. At the end of the treatment, the mean increase in hemoglobin level in the treated patients was found to be 1.57, 2.22, and 2.92 g/dL in 100, 150, and 200 mg treatment arms, respectively, while the change in the placebo group was 0.46 g/dL. The response rate for hemoglobin rise (>1g/dL) was 66% in 100 mg, 75% in 150 mg, and 83% in 200 mg treatment arms, versus 23% in placebo arm, respectively. Total iron binding capacity (TIBC) was found to be increased in all treatment groups at the end of treatment. The transferrin saturation (TSAT) was decreased in 150 mg and 200 mg treatment arms. LDL levels were decreased in 150 mg treatment arm. Hepcidin levels were significantly decreased in all the desidustat treatment arms. The increase in the EPO levels was observed from 4 h to 24 hours post-dose in the first week as well as in the last week of the treatment.

A dose-related increase in Cmax and AUC0–t was observed in desidustat arms after single and multiple doses, and no accumulation was observed after repeated dosing for six weeks. The mean half-life of 6.56–13.79 h observed after first dose was found to be 6.20–8.70 h in the sixth week of the treatment (45).

Treatment emergent adverse events (TEAE) were lesser in the treatment arms than in the placebo arm. TEAEs were mainly abdominal pain, vomiting, and headache, and majority of TEAEs were mild. No patient was discontinued from the trial due to AE, and no deaths occurred during the trial. The number of patients experiencing AEs during the trial do not significantly differ (p > 0.05) in the treatment and placebo arms. No safety issues were observed in terms of laboratory values, vitals, and liver function tests (45).

#### 3.4.2 Desidustat was noninferior to darbepoetin or epoietin alfa in phase 3 trial

In phase 3 study, desidustat was tested against darbepoetin. Patients of anemia (n=588) due to CKD without dialysis need, with baseline hemoglobin of 7.0-10.0 g/dL were randomized to receive either desidustat 100 mg (oral tablets thrice a week) or biosimilar darbepoetin (subcutaneous injection, 0.75 μg/kg once in two weeks) for 24 weeks (46).

Desidustat treatment caused a hemoglobin rise of 1.95 g/dL from the baseline, while the increase observed in darbepoietin group was 1.83 g/dL in the darbepoetin, confirming that desidustat treatment was noninferior to darbepoietin, with more responders in desidustat group for hemoglobin rise than in the darbepoietin group. Also, desidustat caused a significant decrease in hepcidin and low-density lipoprotein, and did not change the vascular endothelial growth factor after the treatment, at the tested dose (47). Majority of the AEs were mild, unrelated with the treatment, and resolved, and there were no treatment-related deaths. The treatment emergent adverse event hypertension had more incidents in the darbepoetin treated group (5.78%) compared to the desidustat group (1.70%).

In dialysis-dependent patients of CKD phase 3 study of desidustat was conducted across 38 centers in India (47). A total of 392 patients with clinical diagnosis of anemia due to CKD with dialysis need (Erythrocyte Stimulating Agent [ESA] naïve or prior ESA users) and with baseline hemoglobin levels of 8.0-11.0 g/dL (inclusive) were randomized in a 1:1 ratio to receive either desidustat oral tablets (thrice a week) or epoetin alfa subcutaneous injection for 24 weeks to maintain a hemoglobin level of 10-12 g/dL. The primary endpoint was to assess the change in the hemoglobin level between the desidustat and the epoetin alfa groups from the baseline to evaluation period week 16-24. The key secondary efficacy endpoint was the number of patients with hemoglobin response. The change in hemoglobin from the baseline to week 16-24 was 0.95 g/dL in the desidustat group and 0.80 g/dL in the epoetin alfa group, which met the prespecified noninferiority margin. The number of hemoglobin responders was significantly higher in the desidustat group (106 [59.22%]) when compared to the epoetin alfa group (89 [48.37%]) (p = 0.0382). The safety profile of the desidustat oral tablet was comparable with the epoetin alfa injection. There were no new risks or no increased risks seen with the use of desidustat compared to epoetin alfa. Thus, at the tested dose, desidustat profile is comparable in safety with epoetin alfa.

## 4 Desidustat: favorable profile for CKD-induced anemia treatment

### 4.1 Desidustat is potent PHD inhibitor and effective HIF stabilizer

Most of the PHD inhibitors show nanomolar potency in PHD binding assays, though from translational mechanism perspective, HIF stabilization is a rate-limiting target engagement tool to assess the efficacy of any PHD inhibitor (37). Daprodustat (48) roxadustat (49), vadadustat (50), and desidustat (37) demonstrate HIF stabilization at micromolar potency in cell-based assays. A notable difference is the suboptimal effect (Emax) of daprodustat, which may have been translated in lesser EPO release caused by daprodustat in preclinical as well as clinical studies (51). On the other hand, the reticulocyte count, which is the hallmark of erythropoiesis, was found to be significantly increased with desidustat, as compared to vadadustat (52).

### 4.2 Desidustat shows clinical effect without sudden rise in hemoglobin and without off target side effects

In CKD patients with anemia chronic inflammation and comorbidities are prominent. Desidustat has shown efficacy for improving hemoglobin at the same dose in anemia models of inflammation (PGPS-induced) or nephrectomy-induced CKD (38, 39, 42). PHD inhibitors have been reported to have toxicities related to accelerated pharmacology, arising out of elevated hemoglobin. However, no off-target toxicities observed following desidustat treatment (27, 44–47), as against more cancer-related death or tumor progression events and more esophageal or gastric erosion events in the daprodustat treatment (53). Reports indicated a higher portion of nondialysis-CKD patients and dialysis dependent-CKD patients treated with roxadustat experienced vascular access thrombosis compared with controls (28, 54). Vadadustat failed to meet noninferiority criteria in Major Adverse Cardiovascular Events (MACE) trial in the non-dialysis patient population, showed increased risk of thromboembolic events, driven by vascular access thrombosis in dialysis patients, and the risk of drug-induced liver injury (52). The roxadustat preclinical toxicity study indicated the side effects such as chronic or chronic active valve inflammation, increased production, myxomatous degeneration, and fibrinous thrombus in the heart; glandular mucosal erosion, ulcer, and inflammation in the stomach (54). Thus, appearance of off-target or severely increased thrombotic or gastrointestinal side effects in the trials were the major reasons for the decrease in the benefit/risk profile of the currently used drugs of PHD inhibitor class. Compared to other PHD inhibitors, desidustat treatment, either in nondialysis patients or in dialysis-dependent patients is noninferior to either epoetin alpha or darbepoietin (46, 47), without any serious side effect related to tumor progression, gastrointestinal trouble, or thrombosis. Significant enough, it showed a prominent decrease in hepcidin (45) levels.

The FDA review highlighted that the appearance of the thrombotic or hematological side effects of roxadustat could be related to the rise in the hemoglobin in the first eight weeks of trial, especially in the nondialysis patients (55). The increase in hemoglobin in the nondialysis patients, within eight weeks of treatment with roxadustat, was, indeed higher than the comparator ESA treatment (56), while it was like ESA in case of daprodustat (57) and desidustat (46). However, a major difference appears to be significantly higher efficacy in terms of hemoglobin rise by desidustat treatment, compared to daprodustat (36, 46). Thus, at tested doses and duration, desidustat seems to be effective with optimum increase in erythropoietin levels in preclinical and clinical settings, without sudden increase in hemoglobin within eight weeks of treatment in clinic, and it is devoid of any serious side effects, especially the off-target thrombotic or gastrointestinal side effects in up to phase 3 clinical trials (46, 47).

### 4.3 Desidustat has a favorable pharmacokinetics

Most PHD inhibitors approved as on now, have a common pharmacophore containing glycinamide with carboxylic group at the terminal. Majority of these molecules have hydrophobic rings attached directly to central core or through spacer groups. Among these inhibitors, daprodustat differs in having sp3 rich cyclohexyl ring; however this has led to the formation of several polyoxygenated metabolites, which is mainly mediated by CYP2C8 and secondarily by CYP3A4 (57, 58). Vadadustat forms GSH-adduct during metabolism (52). GSH adduct formation can be associated with drug-induced liver, skin, and hematopoietic toxicity of many drugs leading to clinical toxicities (59). On other hand, Desidustat is comparatively a flat molecule and has relatively less hydrophobic sites for such metabolism (29). This is reflected in presence of minimally formed (<10%) metabolites in clinical studies.

Desidustat treatment is recommended once every alternate day, while vadadustat or daprodustat are prescribed for once daily administration. Desidustat administration does not induce any reactive or active metabolites and based on *in vitro* data, no clinically relevant drug-drug interactions can be predicted that are mediated by CYPs or transporter interactions. Additionally, no drug interaction is expected with phosphate binders and oral iron preparation (as no significant alteration in aqueous solubility of desidustat in the presence of phosphate binders or multivalent cations) (27). **Table 1** shows the summary of the pharmacokinetic data of selected PHD inhibitors.

**Table 1:** Pharmacokinetics of selected PHD inhibitors.

Drug	Dose	Dosing schedule	Oral absorption	Metabolism	Excretion in urine	Elimination half-life	Interaction with human transporters	Potential drug interaction
Roxadustat	0.7 to 2.5 mg/kg	Twice a week	• 40-80% (%F) • T_max_:2 to4 h (range) • no drug exposure accumulation upon repeated administration • (3 times a week); • no PK differences between peritoneal dialysis & hemodialysis patients	Mainly metabolized via CYP2C8 and UGT1A9	About 1%	8 to 21 h	Substrate of BCRP, OATP1B1, OAT1 and OAT3 Inhibitor of BCRP and OATP1B1	Clinical drug interactions with phosphate binders, multivalent iron preparations, HMG-CoA reductase inhibitor, probenecid and gemfibrozil
Daprodustat	2-25 mg	Once daily	• 65% (%F) • T_max_:1 to 4 h (range) • no change in PK with once-daily repeated administration	Mainly metabolized by CYP2C8 and lesser extent with CYP3A4	0.05%	1 to 7 h	Substrate of BCRP Inhibitor of OATP1B1 and OATP1B3	Potential drug interaction with CYP2C8 inhibitor and inducer
Vadadustat	300-600 mg	Once daily	• greater than 75% (%F) • T_max_: 1 to 6 h (range) • no drug exposure accumulation following once-daily repeated dosing	Primarily metabolized via UGTs (UGT1A1, UGT1A7, UGT1A8, and UGT1A9)	Less than 1%	4 to 7 h	Vadadustat: Substrate and inhibitor of BCRP, OAT1, OAT3, and OATP1B1 O-glucuronide metabolite: Substrate of MRP2, OAT1B3, OAT3 and inhibitor for OAT1, OAT3	Drug interactions with iron-containing preparations, probenecid and DDIs mediated via human drug transporters (BCRP and OAT3)
Desidustat	100- to 150 mg	Twice a week	• Predicted high oral bioavailability • T_max_: 1 to 5 h (range) • no drug exposure accumulation with an alternate day dosing schedule	Less susceptible for metabolism via CYP450 enzymes (metabolically stable in human liver microsomes and human hepatocytes; minor (<10%) hydroxylation and hydroxyl-glucuronide conjugate metabolite metabolites identified in the humans	28-41%	7 to 11 h	Not a substrate of P-gp or BCRP under the mild acidic apical pH (5.5) Inhibitor of BCRP No significant interaction with OATP1B1, OATP1B3, OAT1, and OCT2, except OAT3	No significant clinically relevant DDIs predicted which are mediated via CYPs and human drug transporters based on primary *in vitro* data. No significant drug interactions are predicted with phosphate binders or oral iron preparations as no significant alteration in aqueous solubility of desidustat in the presence of phosphate binders or multivalent cations

### 4.4 Desidustat is effective in EPO refractory state

Some anemic chronic kidney disease (CKD) patients are refractory to erythropoietin (EPO) effects due to inflammation, deranged iron utilization, and generation of EPO antibodies. Desidustat inhibited EPO-resistance caused by rhEPO treatment in preclinical studies, decreased hepcidin, IL-6, IL-1β, and increased iron and liver ferroportin (36). This treatment also reduced EPO requirement and anti-EPO antibodies and maintained normal hemoglobin levels after cessation of rhEPO treatment. A substantial and sustained improvement in hemoglobin levels was also observed in a clinical case of pure red cell aplasia (PRCA) emphasizing the crucial role of desidustat intervention in EPO-induced PRCA cases (59). Thus, desidustat can treat EPO resistance via improved iron utilization and decreased inflammation.

## 5 Potential of desidustat for safer treatment of CKD-induced anemia

HIF-PHD inhibitors have demonstrated their therapeutic use in managing anemia in CKD patients, since the first approval and launch of roxadustat in China and Japan. This was followed by approvals of vadaustat and daprodustat in Japan, and of daprodustat and vadadustat in USA for the dialysis patients who were on dialysis for at least four months. An ideal HIF-PHD inhibitor should be devoid of the potential liabilities such as drug-induced liver injury, higher incidences of cancer, easophageal erosions, etc. CKD patients are usually treated with multiple drugs including statins, anti-hypertensives, anti-diabetics, clopidogrel, iron supplements or phosphate binders (60). Hence, it is important for an ideal HIF-PH inhibitor to demonstrate minimal potential for drug-drug interactions. These ideal characteristics of a HIF-PHD inhibitor with low risk/benefit ratio may provide a better therapeutic index (60–64). On this background, desidustat profile looks promising. Further long-term clinical and post-marketing studies may prove useful to realize the potential of desidustat.
